# Letter from Washington—Ninth International Medical Congress

**Published:** 1887-09

**Authors:** 


### CORRESPONDENCE.

### THE NINTH INTERNATIONAL MEDICAL CONGRESS.

(Letter from Washington.)

THE ARLINGTON, WASHINGTON, D. C., Sept. 9, 1887.

*Editor Daniel's Texas Medical Journal:*

The amount of work done at this meeting of the Congress is wonderful. There is the general session from 10 to 11 a. m.—and from 11 a. m. to 1 p. m., and again from 3 to 6 p. m. the sections meet. The published Transactions will fill several volumes. It is the most successful session yet held, and is unquestionably the medical event of modern times. Representative men are here from all parts of the civilized world—men of distinction in their profession, and nearly all of whom have contributed papers, and many others who could not get here have sent their papers. The same is true of our home profession. This meeting demonstrates very clearly that all the medical talent of this country is not confined to any geographical limit. The different sections are largely attended, and I have never seen so much earnestness manifested in the discussion of papers. The social features have been exceedingly pleasant, and would have been more varied but for the facts that Washington is at this season almost deserted by those who would extend their hospitality were they here.

The reception by President and Mrs. Cleveland was a very splendid affair. Mr. Cleveland was completely overshadowed by his brilliant wife, and all the doctors, young and old alike, fell in love with her. She is certainly one of the most charming of women; she did not meet one in a cold, formal way, but with a delightful and whole-souled-smile of welcome, would grasp your hand, as though she were meeting some old and dear friend. One of our gallant and chivalrous Texas confreres was heard to say, " Mrs. Cleveland, I am from———, Texas, and it affords me pleasure to express my high appreciation and that of Texas for President Cleveland's wife," which sentiment was heartily endorsed by every one who had the pleasure of her pleasant greeting.

The banquet last night at the Pension Building was really a triumph. The decoration of the immense hall was the most varied and brilliant. Three thousand doctors, most of them in evening dress, and as many ladies, also in evening costume, were assembled there, presenting a picture of beauty and gallantry rarely ever seen. The menu embraced every delicacy, from soup to champagne. However, notwithstanding this fact, the Philadelphia *Press* states that "it was a failure; "the hall was jammed with a crowd fairly well dressed," and "about 1500 bottles of American champagne were opened." That "the Congress was inharmonious;" that "its members were disappointed in its results," and that "the foreign delegates complained," &c. *ad nauseam*. All of which was no doubt instigated by that faction which has done so much to render it a failure; and since they have failed, are now trying to misrepresent, but the effort will be a dead letter, for 3000 missionaries, members of the Ninth International Medical Congress, will go forth to all parts of the civilized world to bear testimony that this has been the most successful Congress that has ever convened, not only in view of the perfect harmony which prevailed, of social enjoyment, and, most important of all, of the amount of scientific work which was offered and accomplished, of which fact the published Transactions will be a lasting evidence.

Texas was well represented, both in point of numbers and ability,—there being present some twenty or twenty-five of our best known physicians. As was to have been expected, the Texas delegates sustained the dignity and honor of the profession in a becoming manner, and "held up their corner" in the discussion of papers in the sections. We recall the presence of Drs. Cupples, of San Antonio, J. W. McLaughlin and T. J. Tyner, of Austin, O. Eastland, of Wichita Falls, H. H. Darr, of Caldwell, Clark, of Moravia, Lockhart, of Chapel Hill, R. B. White, of Ennis, Beall, of Fort Worth, Chilton and Page, of Dallas, Herff, M. K. Taylor and M. K. Taylor Jr., of San Antonio, J. C. Jones, of Gonzales, Frank Paschall, of Chihuahua, Mexico, Paine, West and Randall, of Galveston, Knott, of Goliad, and others. Drs. McLaughlin and others will attend the gynecological meeting in New York next week.

Yours truly,

